# Resource and infrastructure challenges on the RESIST-2 Trial: an implementation study of drug resistance genotype-based algorithmic ART switches in HIV-2-infected adults in Senegal

**DOI:** 10.1186/s13063-021-05902-5

**Published:** 2021-12-18

**Authors:** Dana N. Raugi, Khardiata Diallo, Mouhamadou Baïla Diallo, Dominique Faye, Ousseynou Cisse, Robert A. Smith, Fatima Sall, El Hadji Ibrahima Sall, Khadim Faye, Jean Philippe Diatta, Binetou Diaw, Jacques Sambou, Jean Jacques Malomar, Stephen E. Hawes, Moussa Seydi, Geoffrey S. Gottlieb, for the University of Washington-Senegal HIV-2 Study Group

**Affiliations:** 1 Department of Medicine, Division of Allergy & Infectious Diseases, Center for Emerging & Re-Emerging Infectious Diseases, University of Washington, Seattle, WA USA; 2 Service des Maladies Infectieuses et Tropicales, Ibrahima Diop Mar, Centre Hospitalier National Universitaire de Fann, Universite Cheikh Anta Diop de Dakar, Dakar, Senegal; 3 Centre de Sante de Ziguinchor, Ziguinchor, Senegal; 4 Department of Epidemiology, University of Washington, Seattle, WA USA; 5 Department of Global Health, University of Washington, Seattle, WA USA

**Keywords:** HIV-2, Antiretroviral therapy, Viral suppression, HIV treatment, Point-of-care, COVID-19

## Abstract

**Background:**

Second-line treatment of HIV-2 in resource-limited settings (RLS) is complicated by a lack of controlled trial data, limited availability of HIV-2-active antiretroviral drugs, and inadequate access to drug resistance testing. We conducted an implementation trial of a dried blood spot- (DBS) based, drug resistance genotype-informed antiretroviral therapy (ART) switching algorithm for HIV-2-infected patients in Senegal.

**Methods:**

HIV-2-infected adults initiating or receiving ART through the Senegalese national AIDS program were invited to participate in this single-arm trial. DBS from participants with virologic failure (defined as viral load (VL) > 250 copies/mL after > 6 months on the current ART regimen) were shipped to Seattle for genotypic drug resistance testing. Participants with evidence of drug resistance in protease or reverse transcriptase were switched to new regimens according to a pre-specified algorithm. Participant clinical and immuno-virologic outcomes were assessed, as were implementation challenges.

**Results:**

We enrolled 152 participants. Ten were initiating ART. The remainder were ART-experienced, with 91.0% virologically suppressed (< 50 copies/mL). Problems with viral load testing capability resulted in obtaining VL results for only 227 of 613 (37.0%) participant-visits. Six of 115 participants (5.2%) with VL available after > 6 months on current ART regimen experienced virologic failure, with per-protocol genotypic testing attempted. One additional test was performed for a participant with a VL of 222 copies/mL. Genotypes from three participants showed no evidence of major drug resistance mutations, two showed nucleoside reverse transcriptase inhibitor (NRTI) resistance, one showed both NRTI and protease inhibitor resistance, and one test failed. No integrase inhibitor resistance was observed. Five of six successfully-tested participants switched to the correct regimen or received additional adherence counseling according to the algorithm; the sixth was lost to follow-up. Follow-up VL testing was available for two participants; both of these were virally suppressed (< 10 copies/mL). The trial was terminated early due to the COVID-19 pandemic (which prevented further VL and genotypic testing), planned rollout of dolutegravir-based 1st-line ART, and funding.

**Conclusions:**

The RESIST-2 trial demonstrated that a DBS-based genotypic test can be used to help inform second-line ART decisions as part of a programmatic algorithm in RLS, albeit with significant implementation challenges.

**Trial registration:**

ClinicalTrials.govNCT03394196. Registered on January 9, 2018.

## Background

HIV-2 is a neglected public health problem in West Africa and causes an estimated one to two million infections worldwide [1]. Compared to HIV-1, HIV-2 infection is characterized by lower plasma viral loads (VL), slower CD4 count decline, lower rates of mother-to-child and sexual transmission, longer asymptomatic stage, and slower disease progression [2–6]. However, the majority of HIV-2-infected persons will progress to AIDS and death if left untreated [7, 8], and HIV-2-infected individuals can benefit from antiretroviral therapy (ART) [9].

Effective HIV-2 treatment has significant challenges. Because HIV-2 is intrinsically resistant to many US FDA-approved HIV-1 antiretroviral agents (reviewed in [10]), treatment options are limited. Few clinical trials have been conducted for HIV-2, so existing HIV-2 treatment guidelines are based primarily on in vitro data and observational cohort studies. Prior to the current global roll-out of fixed-dose combination tenofovir-lamivudine-dolutegravir (TLD), most West African national AIDS programs relied primarily on first-line regimens containing ritonavir-boosted lopinavir (LPV/r) plus two nucleoside reverse transcriptase inhibitors (NRTI), due in large part to the need to stock LPV/r as second-line therapy for HIV-1 infection [9]. Guidelines for empiric second-line HIV-2 ART are limited due to lack of controlled trials. Second-line treatment decisions for HIV-2 are also hampered by a lack of HIV-2 VL and drug resistance testing capacity outside of research settings [9, 11–13], which often rely on expensive, infrequent batch-shipping of frozen plasma to Europe or the USA.

We developed and validated a protocol for performing HIV-2 genotypic drug resistance testing of protease (PR), reverse transcriptase (RT), and integrase (IN) from dried blood spots (DBS) [14]. DBS are considered non-hazardous and can be shipped at ambient temperature, allowing for frequent, simple, and inexpensive shipping to HIV-2 reference labs (typically in developed countries) for genotyping in a clinically-actionable timeframe. This technology appears suitable for performing drug resistance testing for patients with plasma VL > 250 copies/mL.

In the RESIST-2 trial, we evaluated a DBS-based, genotype-guided algorithm for second-line treatment of HIV-2 infection, with the overall goal of improving treatment outcomes for HIV-2–infected individuals residing in resource-limited settings (RLS).

## Methods

### Study design and participant population

This single-arm implementation trial of a genotype-driven ART switch algorithm enrolled adults (≥18 years old) infected with HIV-2, who were either already receiving or initiating ART under the Initiative Sénégalaise d’Accès aux Antirétroviraux (ISAARV) program. Individuals who were HIV-1 mono- or HIV-1/HIV-2 dually-seropositive at screening, as well as HIV-2-infected individuals who were not receiving or initiating ART, and HIV-2-infected women who were pregnant or breast-feeding, were excluded. Individuals who had previously received an integrase inhibitor (raltegravir; RAL, or elvitegravir) or darunavir (DRV) were not eligible for study participation. Individuals with a creatinine clearance < 30 or elevated transaminases greater than 2.5 times the upper limit of normal of the assay used were ineligible for safety reasons. The trial had an enrollment target of 150 participants with virologic failure (VF; VL > 250 copies/mL) during follow-up for potential DBS genotyping and algorithmic ART switch. This enrollment target was based on recruitment and virologic failure rates from our previous studies in Senegal [15] and was intended to yield > 100 genotypic results from which to evaluate the algorithm.

We enrolled trial participants at the Service des Maladies Infectieuses et Tropicales (SMIT), Centre Hospitalier National Universitaire de Fann, in the capital city of Dakar, Senegal (beginning in July 2018) and the Centre de Sante de Ziguinchor, the regional capital of the rural region of Casamance, Senegal (beginning in October 2018). The study was conducted according to procedures approved by the US National Institutes of Health, Institutional Review Boards at the University of Washington and the Senegalese National Ethics Committee for Health Research (CNERS). All participants provided written informed consent for study participation. The trial was registered at ClinicalTrials.gov (NCT03394196).

### Study procedures

Participants were screened for HIV infection by serology using combination antibody testing (Determine; Alere), with confirmatory testing using HIV-1/HIV-2 immuno-differentiation assay (SD Bioline HIV-1/2 3.0; Alere, or MultiSure; MP Biomedicals). HIV-2-positive individuals who were receiving or initiating ART were invited to participate. Participants were seen for screening, enrollment, and follow-up visits every 3 months thereafter (or 1-month post-ART initiation/switch where applicable, returning thereafter to quarterly follow-up). Post-VF follow-up was planned for up to 3 years. Participants underwent standardized interviews including demographic characteristics and routine medical histories, including prior ART where applicable, and physical examinations. At each study visit, blood was collected by venipuncture for safety and monitoring labs (blood counts, T cell subsets, and chemistries) using standard methods. Repeat HIV serologic testing and lipid panels were performed annually. Testing for pregnancy, as well as sexually transmitted infections with syndromic management, were performed according to Senegalese guidelines. HIV-2 plasma VL testing was performed in Dakar using the UW HIV-2 assay for the Abbott m2000 platform (Des Plaines, Illinois) (reproducible LOD = 10 copies/mL, absolute LOD = 1 copy/mL) [16]. Viral loads obtained at non-study visits and screening visits before enrollment were not considered eligible for inclusion in the algorithm. Participants with plasma VL > 250 copies/mL after six months or more on their current regimen were defined as having experienced treatment failure and considered eligible for genotypic drug resistance testing; DBS samples from these subjects were shipped to Seattle at ambient temperature via commercial courier. The presence of resistance-associated mutations in the PR, RT, and IN-encoding regions of HIV-2 *pol* was determined via PCR amplification and Sanger sequencing of HIV-2 nucleic acid from the DBS cards as described previously [14]. Drug resistance data were entered on case report forms that included the drug resistance testing results and treatment recommendations per study algorithm, as well as the dates of the study visit, specimen arrival in Seattle, and genotyping completion. Completed forms were emailed back to the study physicians in Senegal.

### Genotyping and switching algorithm

**Fig. 1:**
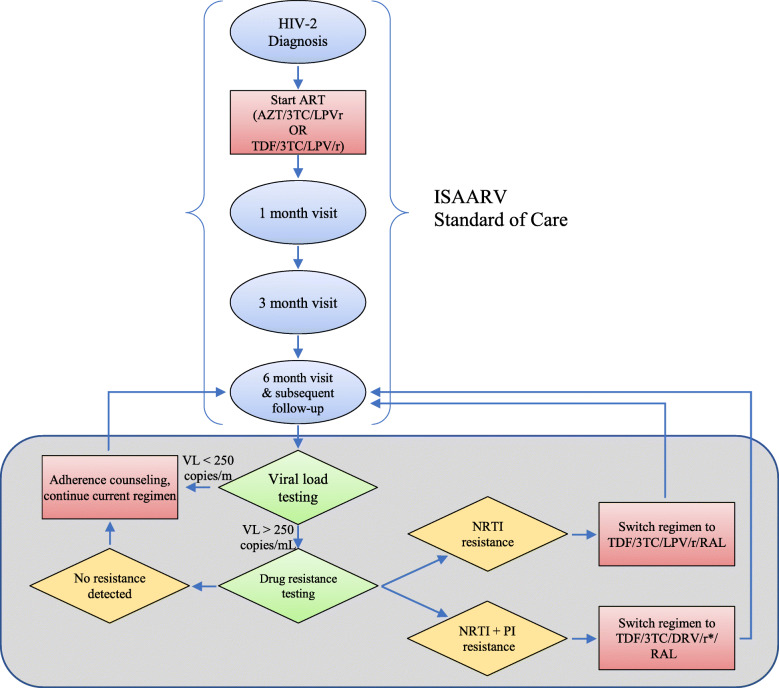
Algorithm for HIV-2 care in the RESIST-2 Trial for genotype-informed second-line therapy in Senegal. Bracketed steps are ISAARV Standard of Care for HIV-2 infection. Steps in gray box represent RESIST-2 Trial of DBS-based drug resistance genotyping to guide second-line therapy decisions. *NRTI* nucleoside reverse transcriptase inhibitor; *PI* protease inhibitor; *3TC* lamivudine; *AZT* zidovudine; *DRV/r* ritonavir-boosted darunavir; *LPV/r* ritonavir-boosted lopinavir; *RAL* raltegravir; *TDF* tenofovir disoproxil fumarate. *Indicates twice-daily dosing

The ART switch algorithm (Fig. 1) used for the trial relied on DBS-based drug resistance testing. Resistance to protease inhibitors (PI), NRTI, and integrase inhibitors (INI) was assessed based on the presence of substitutions V47A, I50V, I54M, and L90M in PR [17–19]; K65R, Q151M, and M184I/V in RT [18–20]; and Y143C, Q148R, and N155H in IN [18, 19, 21], respectively. These amino acid changes are known to be major drug resistance mutations in HIV-2. Participants with no evidence of resistance-associated mutations in RT, PR, or IN were assigned to receive enhanced adherence counseling. Those with evidence of NRTI resistance only were assigned to add RAL to the first-line standard-of-care regimen of LPV/r plus tenofovir and lamivudine (TDF/3TC). Those with evidence of NRTI and PI resistance were assigned to switch to RAL plus twice-daily boosted DRV (DRV/r, 600 mg/100 mg) plus TDF/3TC. There was no defined algorithmic regimen for INI resistance because there have been no reports of pre-treatment HIV-2 INI resistance in Senegal, and all eligible participants were INI-naïve. If a participant experienced virologic failure at two consecutive visits and genotypic testing failed for both, the algorithm called for switching to TDF/3TC, RAL, and twice-daily DRV/r. First-line agents (LPV/r and AZT/3TC or TDF/3TC) were provided under the auspices of ISAARV. Although second-line ARVs are all theoretically available through ISAARV, study drugs were donated by the US manufacturers (DRV; Janssen Pharmaceutica, and RAL; Merck & Co.) or purchased (ritonavir, generic formulation) to mitigate the effects of frequent ISAARV stock-outs of these agents.

### Definitions and analyses

Outcomes for this analysis include the number and percent of participants experiencing virologic failure (VL > 250 copies/mL after > 6 months on ART regimen), drug resistance at virologic failure, and subsequent virologic suppression among those participants who were switched to a new ART regimen based on DBS testing. We also retrospectively assessed and report on challenges to the implementation of a genotype-driven switching algorithm. BMI was calculated and categorized using standard definitions according to the US Centers for Disease Control and Prevention, WHO clinical stage [22], in which history of opportunistic infections is used as a proxy for HIV disease progression, was reported by the clinician. HIV-2 “viral load suppression” was defined as VL < 50 copies/mL to conform to HIV-1 FDA Snapshot definitions. For the purposes of determining time on regimen for genotypic testing eligibility, patients with unknown start dates for their current ART regimen were considered to have initiated the regimen the day prior to study enrollment. Study social workers attempted to contact participants who missed appointments; participants were considered lost to follow-up (LTFU) if there was no contact for > 1 year and were censored at their last visit. Participants who were reported to have died while on study were censored at the date of death. All remaining participants were censored on March 15, 2020, due to COVID-19 pandemic restrictions requiring suspension of study procedures, and the trial was formally terminated by the US NIH/NIAID on May 31, 2020. All statistical analyses were performed in Stata SE 14 (Statacorp; College Station, Texas).

### Sequences

HIV-2 sequences generated for genotypic resistance testing have been deposited in GenBank under the following accession numbers: MT992929-MT992940.

## Results

Unforeseen delays in protocol approvals led to study enrollment commencing two years later than initially planned. The expiration of the study funding period coincided with the anticipated ISAARV roll-out of TLD in 2020 (making the study switch algorithm moot), and the study was officially slated for termination on May 31, 2020, by the sponsor. The US National Institutes of Health/National Institute of Allergy & Infectious Diseases decided to stop new enrollment in mid-2019, which led to limited enrollment and follow-up that was well-short of the planned 3-year follow-up period for any participant. In addition, due to the COVID-19 global pandemic, all study-related follow-up was suspended by March 15, 2020, in accordance with IRB and ethics committee restrictions and could not be re-started before the study was officially terminated.

During the limited study period, we enrolled 152 participants in the trial (Table 1). The majority were female (80.1%), with a median age of 55 years. One hundred and forty-two participants were already receiving ART at enrollment; these subjects had a median CD4 count of 578 cells/μl, and 91.0% had a suppressed VL (< 50 copies/ml). Nine of these participants had no regimen initiation dates available. Ten participants were ART-naïve at enrollment; these participants had a median CD4 count of 332 cells/μl, and 66.7% had a suppressed VL. All participants initiated or were already receiving LPV/r plus two NRTI.

**Table 1:** Baseline characteristics of HIV-2-infected Senegalese adults participating in a drug resistance-based algorithmic ART switching study

	All participants (*n*=152)
Female, number (%)	123 (80.1%)
Age (years), median (IQR)	55 (48–62)
HIV diagnosis year, median (range)	2012 (1995–2019)
ART initiation year, median (range)	2013 (2001–2020)
WHO clinical stage, number (%)	
1	43 (28.5%)
2	30 (19.9%)
3	67 (44.4%)
4	11 (7.3%)
BMI category, number (%)	
Underweight/malnourished (<18.5)	22 (14.5%)
Normal weight (18.5-24.9)	68 (44.8%)
Overweight (25.0-29.9)	41 (27.0%)
Obese (30+)	21 (13.8%)
Participants initiating ART, number (%)	10 (6.6%)
CD4 count^a^ (cells/μL), median (IQR)	332 (173–552)
Plasma viral load^b^ < 50 copies/mL, number (%)	4 (66.7%)
Plasma viral load^c^, log_10_ copies/mL, median (IQR)	1.15 (1.11–2.18)
ART-experienced participants, number (%)	142 (93.4%)
CD4 count^a^ (cells/μL), median (IQR)	578 (359–847)
Plasma viral load^b^ < 50 copies/mL, number (%)	101 (91.0%)
Plasma viral load^c^, log_10_ copies/mL, median (IQR)	1.18 (0.90–2.12)
Baseline ARV regimen	
LPV/r-AZT-3TC	68 (44.7%)
LPV/r-TDF-3TC	84 (55.3%)

*3TC* lamivudine, *AZT* zidovudine, *LPV/r* ritonavir-boosted lopinavir, *TDF* tenofovir disoproxil fumarate

^a^Baseline CD4 count was missing for 2 ART-naïve participants and 16 ART-experienced participants

^b^Baseline viral load was missing for 4 ART-naïve participants and 31 ART-experienced participants

^c^Among those with plasma viral loads > 50 copies/mL

At study suspension on March 15, 2020, 134 participants (88.1%) remained on the study with a median follow-up time of 493 days (IQR: 409-516). Two of those participants remained on study but had not been on their regimen for at least six months and were therefore ineligible for algorithmic ART switch. Fifteen participants (9.9%) had been LTFU, two (1.3%) had died, and one (0.7%) withdrew. Thirteen of the fifteen participants who were LTFU enrolled and never returned for a study visit; the remaining two were seen at 97 and 98 days post-enrollment and were then lost. The participants who died had been on study for 299 and 412 days, and the participant who withdrew had been on study for 528 days. Both participants who died, the participant who was lost, and the two patients who were lost after their first quarterly visit, had been on their ART regimen for at least 6 months and were therefore eligible for algorithmic ART switches.

**Fig. 2:**
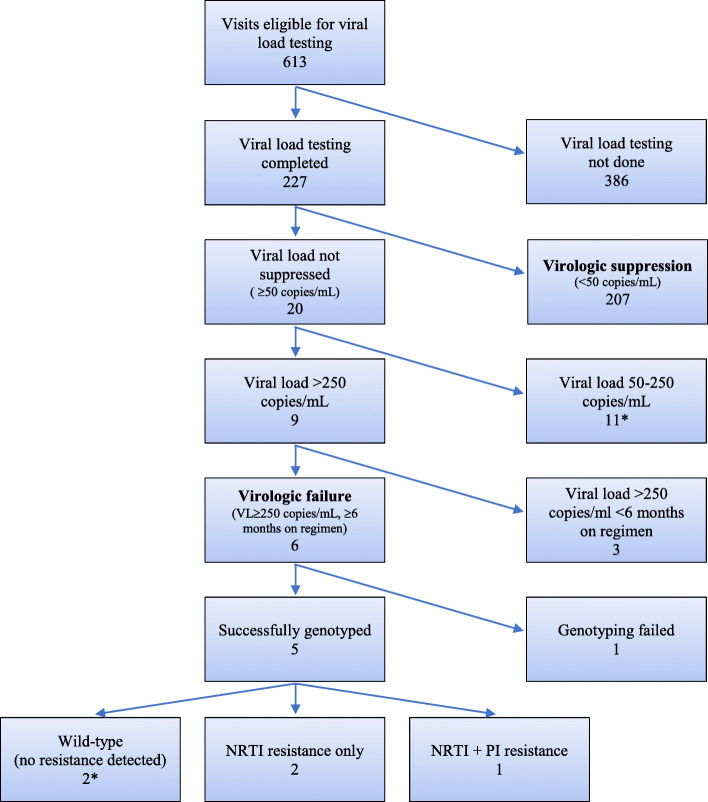
Virologic and drug resistance testing performed in RESIST-2 Trial. *One additional participant had a genotypic test performed with a viral load of 222, which did not meet criteria for virologic failure

Plasma samples from 613 study visits were eligible for HIV-2 RNA VL testing (Fig. 2). Of these, VL testing was performed for 227 (37.0%) specimens representing 126 participants. The remainder were not tested due to problems with the m2000 viral load machine. Of those specimens tested, 207 specimens (91.2%) were considered to be virally suppressed by FDA Snapshot analysis (< 50 copies/mL). The median VL of the remaining 20 specimens was 201 copies/mL (IQR: 114–1085), Nine samples contained HIV-2 VL > 250 copies/mL, and of these, six samples representing six unique participants were collected after > 6 months of ART, meeting protocol-defined criteria for VF drug resistance testing and possible algorithmic ART switching (Table 2). Due to known challenges with obtaining viral loads and the probability of increasing viral load post-failure, drug resistance testing was also performed for an additional participant who had a VL slightly less than the protocol-defined requirement (VL 222 copies/mL).

**Table 2:** Drug resistance genotyping and algorithmic switching results among HIV-2-infected Senegalese adults

Patient number	Viral load (copies/mL)	Visit to results (days)	Visit to switch (days)	RT resistance mutations	PR resistance mutations	Algorithm-specified regimen	Algorithm followed
10	378	56^a^	-^a^	-^a^	-^a^	-^a^	
26	359	58	-	-	-	No change	Yes
57	576	62	119	K65R, M184V	-	3TC, TDF, LPV/r, RAL	Yes
108	730	57	118	M184V	I50V	3TC, TDF, DRV/r, RAL	Yes
124	1439	71	-	-	-	No change	Yes
178	6774	118	153	M184I	-	3TC, TDF, LPV/r, RAL	Yes
569	222	126	-	-	-	No change	^b^

*3TC* lamivudine, *DRV/r* ritonavir-boosted darunavir, *TDF* tenofovir disoproxil fumarate, *LPV/r* ritonavir-boosted lopinavir, *RAL* raltegravir

^a^Test failed, no PCR products were obtained after using all available nucleic acid. In the absence of subsequent viral load testing and genotypic testing, this participant remained on existing regimen with enhanced adherence counseling

^b^Participant was lost to follow-up after genotyped visit

For the seven participants that received genotypic drug resistance testing, the median time from a study visit to genotype report was 62 days (range 56–126; Table 2). One genotypic resistance test from a participant with a VL of 378 copies/mL failed due to an inability to amplify PCR products from the DBS sample, no viral load testing was available at any subsequent study visit and the participant remained on their LPV/r-based regimen.

HIV-2 sequences from three participants had no detectable major resistance-associated mutations in PR, RT, or IN (Table 2). Two of these three participants (#26 and 124) received enhanced adherence counseling at their next study visit; the third (#569), who had genotyping performed despite being under the protocol-defined VL limit, was lost to follow-up immediately after the genotyping visit. No follow-up VL data were available for the two participants who received enhanced adherence counseling.

Three participants’ genotypes revealed drug resistance-associated changes (Table 2). One participant had RT change M184I (the precursor to M184V), one participant had RT changes K65R and M184V, and one participant had RT change M184V and PR change I50V. No participants had evidence of resistance to INI. The three participants with observed drug resistance all switched to the correct algorithmic regimen. Two participants (#57 and 108) who switched regimens had undetectable VL (< 10 copies/ml) at their next follow-up visit but had no subsequent VL data available; no additional VL testing was available for the third participant (#178).

Once the study was underway, significant challenges to algorithm implementation were noted. Most critically, the SMIT Abbott m2000 system was functional for only 251 days out of the 617-day study period (40.7%). The m2000 requires proprietary consumables, and unexpectedly lengthy delays were encountered in receiving these supplies. In addition, the m2000sp (nucleic acid extraction unit) required two repairs, and a replacement uninterruptable power supply and voltage converter had to be obtained. These repairs contributed more than four months of machine down-time. Finally, when the roof at SMIT leaked during a major rainstorm in September 2019, the m2000rt (real-time PCR machine) optics were irreparably damaged, 6 months prior to the cessation of follow-up. Even if the machine had been fully operational, dry ice or liquid nitrogen on which to ship frozen specimens from Ziguinchor to Dakar was only sporadically available, hindering timely shipments.

## Discussion

Several studies have used DBS-based testing to perform population-level assessments of the frequency of drug resistance in HIV-1 [23–25] or to analyze ART outcomes in HIV-1-infected patients [26–28]. However, to our knowledge, no studies have attempted to use DBS testing to prospectively inform treatment decisions for HIV-1 or HIV-2-infected individuals in a programmatic setting. We recently described the development and use of a DBS-based method for drug resistance testing of HIV-2-infected patients receiving ART [14, 29]. Here, we evaluated this testing strategy, when used with a US-based HIV-2 reference research laboratory, for use in guiding ART switching decisions within the context of a programmatic, second-line treatment algorithm in Senegal, West Africa.

The conclusions that can be drawn from this trial itself are limited. Although we had planned to perform genotypic testing with possible algorithmic switch for up to 150 participants with VF, the decision was made by NIH/NIAID in mid 2019 to stop new enrollment. This decision was based on several factors, but ultimately was a result of the planned global rollout of TLD. The original estimates of virologic failure were based on our 13-year study of ART for HIV-2 in Senegal [15] but programmatic ART during that study included less-effective regimens including indinavir-based and atazanavir-based, as well as infrequently-available RAL and DRV. The sporadic use of RAL and DRV not only likely contributed to VF rates but also rendered those participants ineligible for the current study. As a result, higher-than-expected rates of virologic suppression among eligible participants meant that a much larger number of participants would have been needed, and recruitment of new study participants was hampered by fewer-than-anticipated newly-diagnosed individuals and less enthusiasm from outside clinic providers for referring patients likely experiencing treatment failure. Delays in initiating the trial and slower-than-anticipated enrollment made achieving the number of participants necessary to obtain 150 participants experiencing VF unrealistic within the original funding period. Finally, ISAARV’s planned switch of all Senegalese HIV-infected patients receiving treatment under their auspices to TLD in 2020 would have effectively eliminated the study population. This made continuing enrollment futile.

Encouragingly, most participants in this study were doing well on their existing ART regimen, with only six of 115 participants (5.2%) who had been receiving their current regimen for > 6 months experiencing virologic failure. However, importantly, individuals who had previously received second-line DRV or RAL through the national program were excluded from this study. Although we observed high rates of virologic suppression within our cohort, those experiencing virologic failure may benefit from the implementation of programmatic genotype-based switching algorithms in order to preserve therapeutic options. This is particularly true given the relatively small number of ARVs, particularly HIV-2-active agents, available through West African national AIDS programs. Of the six participants who received genotypic drug resistance testing in this study, half had no identified drug resistance mutations, potentially indicative of non-adherence rather than treatment failure. In this population, switching may yield little benefit beyond that gained by enhanced adherence counseling. Among those with detected drug resistance, both the participant who added RAL to their existing regimen as well as the participant who switched to RAL and twice-daily DRV/r had VL < 10 copies/mL at the next study visit, demonstrating that the genotype-guided switch led to subsequent viral suppression during short-term follow-up. In RLS, drug resistance testing in-country is generally limited in availability for HIV-1, and not available for HIV-2. However, this study demonstrates that drug resistance genotyping of HIV-2 in reference labs in developed countries can be accomplished in a clinically-actionable timeframe—3 to 6 months, the time between standard-of-care HIV care visits—through the use of DBS.

Although we have demonstrated that HIV-2 DBS genotyping is possible, the challenges to implementing it are enormous, in large part due to the requirement for HIV-2 VL testing. The technical expertise, supply chain, and infrastructure requirements to keep any real-time PCR platform running in RLS are considerable, and commercially-available HIV-2 VL options are limited. Although the BioCentric Generic HIV-2 Charge Virale (BioCentric, France) [30] kit has recently been developed to work on any real-time PCR platform and with non-proprietary consumables, to our knowledge, neither it nor the point-of-care Abbott m-PIMA Analyser HIV-1 and HIV-2 assay have been widely implemented, and research assays are rare. The Abbott m2000 machine at SMIT, which runs the only clinically-available HIV-2 VL assay in the country of which we are aware, was non-functional for more than half of the study period, and remained that way for more than 18 months after study termination. Even relatively common lab equipment owned and maintained by the Senegalese national program was difficult to keep operational and fully supplied due to funding and supply chain issues. For example, the FACSCount machine used for determining CD4 counts for the entire city of Ziguinchor became inoperable in March 2019 and was still not repaired at the end of the study a year later. Indeed, one study evaluating the programmatic feasibility of DBS-based virologic follow-up of HIV-1-infected patients in the Democratic Republic of the Congo noted that the primary obstacle to implementing this technology was the interruption of funding to the national lab performing the work [31]. Complicating the potential use of centralized reference laboratories for HIV-2 VL testing, dry ice and/or liquid nitrogen for dry shippers is not always readily available for shipping frozen plasma. Without plasma VL to trigger reflex genotyping, drug resistance testing from DBS is not feasible. Unfortunately, genotyping based on decreasing CD4 counts alone also has drawbacks, as it is unknown what CD4 decrease over a given timeframe would serve as a reliable proxy for sufficiently high VL to prompt genotypic testing.

Recent advances in point-of-care VL testing technologies represent a potentially beneficial development in the quest to improve clinical care of people living with HIV in RLS [32]. These devices, which include HIV-2 as well as HIV-1 VL testing, eliminate many of the infrastructure and technological expertise requirements that proved to be the downfall of HIV-2 VL testing in this study. In addition, because these devices were designed to be used at point-of-care in RLS, they eliminate the need to ship frozen specimens altogether.

Beyond the challenges of implementing HIV-2 DBS-based genotypic testing in RLS, the effects of the COVID-19 pandemic on this trial were significant and warrant mention. Study follow-up was discontinued prematurely due to the disruption of routine healthcare and research activities, with only medication refills and urgent or emergent medical care being permitted. Secondary effects of the pandemic impacted other research plans as well. COVID-related border closures, travel restrictions, and funding gaps led to myriad problems: planned repairs of the m2000 platform could not be executed, which resulted in an inability to perform additional concurrent or retrospective VL testing to identify additional participants experiencing VF. Samples could not be shipped, study staff were furloughed, and routine maintenance of laboratory infrastructure suffered, resulting in the failure of a study freezer. With return to routine medical care only becoming possible in December 2020, the opportunity cost of pandemic-related shutdowns is likely to be significant both to research as well as to the patients who stand to benefit from this work.

## Conclusions

In this study, we demonstrate that HIV-2 genotypic drug resistance testing to guide algorithmic second-line ART decisions is possible, albeit with significant implementation hurdles. New and developing technologies, including point-of-care VL testing devices, may simplify implementation in the future and could lead to improvements in clinical care for HIV-2-infected patients in RLS.

## Data Availability

The datasets used during the current study are available from the corresponding author on reasonable request. HIV-2 sequences generated for genotypic resistance testing have been deposited in GenBank under the following accession numbers: MT992929-MT992940.
